# ^89^Zr-labeled ImmunoPET targeting the cancer stem cell antigen CD133 using fully-human antibody constructs

**DOI:** 10.1186/s13550-024-01091-9

**Published:** 2024-03-18

**Authors:** Kevin Wyszatko, Melissa Chassé, Nancy Janzen, Luis Rafael Silva, Luke Kwon, Teesha Komal, Manuela Ventura, Chitra Venugopal, Sheila K. Singh, John F. Valliant, Saman Sadeghi

**Affiliations:** 1 Department of Chemistry and Chemical Biology, McMaster University, Hamilton, ON Canada; 2 Institute of Medical Science, Temerty Faculty of Medicine, University of Toronto, Toronto, ON Canada; 3 Spatio-Temporal Targeting and Amplification of Radiation Response Innovation Centre (STTARR), University Health Network, Toronto, ON Canada; 4 Centre for Discovery in Cancer Research, McMaster University, Hamilton, ON Canada; 5 Department of Surgery, McMaster University, Hamilton, ON Canada

**Keywords:** ImmunoPET, Cancer Stem cells, CD133, Zirconium-89, Antibody, Antibody fragment

## Abstract

**Background:**

Cancer stem cells play an important role in driving tumor growth and treatment resistance, which makes them a promising therapeutic target to prevent cancer recurrence. Emerging cancer stem cell-targeted therapies would benefit from companion diagnostic imaging probes to aid in patient selection and monitoring response to therapy. To this end, zirconium-89-radiolabeled immunoPET probes that target the cancer stem cell-antigen CD133 were developed using fully human antibody and antibody scFv-Fc scaffolds.

**Results:**

ImmunoPET probes [^89^Zr]-DFO-RW03_IgG_ (CA = 0.7 ± 0.1), [^89^Zr]-DFO-RW03_IgG_ (CA = 3.0 ± 0.3), and [^89^Zr]-DFO-RW03_scFv − Fc_ (CA = 2.9 ± 0.3) were radiolabeled with zirconium-89 (radiochemical yield 42 ± 5%, 97 ± 2%, 86 ± 12%, respectively) and each was isolated in > 97% radiochemical purity with specific activities of 120 ± 30, 270 ± 90, and 200 ± 60 MBq/mg, respectively. In vitro binding assays showed a low-nanomolar binding affinity of 0.6 to 1.1 nM (95% CI) for DFO-RW03_IgG_ (CA = 0.7 ± 0.1), 0.3 to 1.9 nM (95% CI) for DFO-RW03_IgG_ (CA = 3.0 ± 0.3), and 1.5 to 3.3 nM (95% CI) for DFO-RW03_scFv − Fc_ (C/A = 0.3). Biodistribution studies found that [^89^Zr]-DFO-RW03_scFv − Fc_ (CA = 2.9 ± 0.3) exhibited the highest tumor uptake (23 ± 4, 21 ± 2, and 23 ± 4%ID/g at 24, 48, and 72 h, respectively) and showed low uptake (< 6%ID/g) in all off-target organs at each timepoint (24, 48, and 72 h). Comparatively, [^89^Zr]-DFO-RW03_IgG_ (CA = 0.7 ± 0.1) and [^89^Zr]-DFO-RW03_IgG_ (CA = 3.0 ± 0.3) both reached maximum tumor uptake (16 ± 3%ID/g and 16 ± 2%ID/g, respectively) at 96 h p.i. and showed higher liver uptake (10.2 ± 3%ID/g and 15 ± 3%ID/g, respectively) at that timepoint. Region of interest analysis to assess PET images of mice administered [^89^Zr]-DFO-RW03_scFv − Fc_ (CA = 2.9 ± 0.3) showed that this probe reached a maximum tumor uptake of 22 ± 1%ID/cc at 96 h, providing a tumor-to-liver ratio that exceeded 1:1 at 48 h p.i. Antibody-antigen mediated tumor uptake was demonstrated through biodistribution and PET imaging studies, where for each probe, co-injection of excess unlabeled RW03_IgG_ resulted in > 60% reduced tumor uptake.

**Conclusions:**

Fully human CD133-targeted immunoPET probes [^89^Zr]-DFO-RW03_IgG_ and [^89^Zr]-DFO-RW03_scFv − Fc_ accumulate in CD133-expressing tumors to enable their delineation through PET imaging. Having identified [^89^Zr]-DFO-RW03_scFv − Fc_ (CA = 2.9 ± 0.3) as the most attractive construct for CD133-expressing tumor delineation, the next step is to evaluate this probe using patient-derived tumor models to test its detection limit prior to clinical translation.

**Supplementary Information:**

The online version contains supplementary material available at 10.1186/s13550-024-01091-9.

## Background

Tumorigenic cancer stem cells (CSCs) demonstrate increased resistance to chemo- and radiotherapy treatments and these properties suggest a major role played by CSCs in driving cancer recurrence [1, 2]. Elevated CSC biomarkers can be found in aggressive, treatment-refractory tumors including glioblastoma, breast, lung, and pancreatic cancer, and tumors rich in CSCs are more likely to recur and lead to worse patient outcomes [3–7]. For these reasons, significant interest has been garnered towards the development of molecularly targeted therapies that can eliminate CSCs in tumors, such as immunotherapies, antibody-drug conjugates, and targeted radionuclide therapy [8–10]. In contrast to CSC targeted molecular therapies, there has been comparatively less work done around the creation and evaluation of CSC targeted molecular imaging agents, either as companion diagnostics or standalone imaging agents. Imaging CSCs in tumors, which make up less than 1% of the tumor cell population, is challenging and may be best addressed through the use of high sensitivity imaging modalities like positron emission tomography (PET) using probes with potent (low-nanomolar) binding affinity for CSC-selective antigens [11].

CD133 is a cell-surface glycoprotein overexpressed by CSCs in a variety of tumors making it a promising molecular target for CSC-targeted therapies and molecular imaging [12]. Previous reports on CD133 imaging have labeled a humanized anti-CD133 antibody, AC133.1, with positron emitting radioisotopes copper-64 and zirconium-89 to successfully image CD133 expressing human colorectal and brain tumor xenograft mouse models [13, 14]. Other reports have used a zirconium-89 labeled CD133-targeted murine antibody derived from hybridoma clone B7 to image cell-line derived and orthotopic patient derived models of lung cancer [15, 16]. While these reports have established the feasibility of CD133 imaging using antibody constructs and advanced mouse models, further translation of these probes to the clinical setting may require further humanization due to potential immunogenicity of non-human antibody scaffolds [17]. Furthermore, glycosylated Fc-regions present on these previously reported anti-CD133 antibody PET probes could induce Fc-effector immune responses that are undesired for molecular imaging applications [18]. Minimally immunogenic, fully human antibody constructs with aglycosylated Fc-regions would better facilitate clinical translation of CD133 targeted companion diagnostic imaging probes.

Here we report the preparation and preclinical evaluation of a CD133-targeted imaging probe based on a fully human anti-CD133 antibody and an aglycosylated scFv-Fc antibody fragment with the same binding domain, both radiolabeled with zirconium-89 for PET. The immunoPET probes were characterized for radiochemical yield and purity, immunoreactive fraction, and binding affinity prior to in vivo experiments. Tumor uptake and in vivo specificity of the probe were established through biodistribution and imaging studies using a CD133-expressing tumor xenograft mouse model.

## Methods

### Materials

All reagents were ACS grade, and where indicated, reagents were > 99.995% trace metal-free (TMF-grade). Isothiocyanate-functionalized deferoxamine (*p*-NCS-Bn-DFO) was purchased from CheMatech (C121). N-2-hydroxyethylpiperazine-N-2-ethane sulfonic acid (HEPES) was purchased from ThermoFisher (J16926.A1). Oxalic acid (TMF-grade) (658,537), sodium carbonate (Na_2_CO_3_) (223,530), ethylenediaminetetraacetic acid (EDTA) (TMF-grade) (60-00-4), Tween80 (P1754), and bovine serum albumin (BSA) (9048-46-8) were purchased from Millipore Sigma. Ultrapure 18.2 Ω water was used to prepare all buffers. Animal injections were formulated in a solution of 0.9% NaCl with 0.01% Tween80 buffered to pH 6.8 with sodium acetate (0.01 M) (ABST buffer). [^89^Zr]-oxalate was purchased from 3D Imaging (Little Rock, AR). The fully human anti-CD133 monoclonal antibody (RW03_IgG_) and scFv-Fc (RW03_scFv-Fc_) were provided by Dr. Jason Moffat (University of Toronto) under a research agreement with Century Therapeutics Canada. The Fab used for RW03_IgG_ and the scFv used for RW03_scFv-Fc_ were synthesized via the Cellectseq method from phage-display libraries, using CD133-transfected HEK293 cells and the parental HEK293 cells for positive and negative selection, respectively [19].

Size exclusion chromatography-high performance liquid chromatography (SEC-HPLC) characterization was performed on Waters 1525 Binary HPLC using a Yarra 3 μm Sect. 3000 (300 × 7.8 mm, Phenomenex, 00 H-4513-E0), with phosphate buffer (0.05 M, pH 6.8) as the mobile phase. Samples were analyzed using a Waters 2489 UV/Vis detector (λ = 280 nm). Chromatograms were recorded and processed on Empower 2 software (Waters). Radio-iTLC studies were spotted on iTLC-SG glass microfiber chromatography paper (SGI0001, Agilent Technologies) plates using a solution of EDTA (50 mM, pH 7, adjusted with NaOH) in water as the eluent and analyzed using a Bioscan AR-2000 Imaging Scanner.

#### Conjugation of RW03_scFv-Fc_ with *p*-NCS-Bn-DFO

The CD133-binding antibody, RW03_IgG_ (glycosylated), and CD133-binding antibody scFv-Fc fragment, RW03_scFv − Fc_ (aglycosylated), were conjugated with *p*-NCS-Bn-DFO using a previously published procedure [20]. In a typical reaction, 1–2 mg of the scFv-Fc was exchanged into sodium bicarbonate buffer (0.2 M, pH 9.3) using SEC (PD-10 column, GE Healthcare) followed by concentration to 5 mg/mL using centrifugal filtration (30 kDa cutoff) (Amicon Ultra-15 Centrifugal Filter Unit, Millipore Sigma). To the IgG solutions was slowly added either a 5- or 30- fold molar excess of *p*-NCS-Bn-DFO (10 mg/mL, dissolved in DMSO) and to the scFv-Fc solution was slowly added a 10-fold molar excess of *p*-NCS-Bn-DFO. Each reaction was gently stirred at room temperature (RT) for 4 h (final DMSO concentration < 5% v/v). Products were purified using SEC into phosphate buffer (0.02 M, pH 7.4) and frozen at -80^o^C until future use.

#### MALDI-MS

Matrix-Assisted Laser Desorption Ionization Mass Spectrometry (MALDI-MS) (Bruker UltrafleXtreme TOF/TOF) was performed in positive mode to determine the average chelator-to-antibody scFv-Fc ratio (C/A). Samples were concentrated by centrifugal filtration to 5 mg/mL and then diluted to 1 mg/mL with phosphate buffer (0.02 M, pH 7.4) and diluted 5-fold with a saturated solution of sinapinic acid in TA30 solvent (30:70 v/v acetonitrile: 0.1% TFA). The chelator-to-antibody ratio was calculated by subtracting the molecular weight of the parent IgG or scFv-Fc (MW_S_) from the conjugate (MW_C_) and dividing by the molecular weight of DFO (753 g/mol) (Eq. 1).

1 $$\eqalign{ C/A & =\frac{\left(\frac{{MW}_{C1}{+MW}_{C2}{+MW}_{C3}}{3}\right)-\left(\frac{{MW}_{S1}{+MW}_{S2}{+MW}_{S3}}{3}\right)}{753} \cr & \pm \frac{\left(\sqrt{{\left({StDev}_{{C}_{1}{C}_{2}{C}_{3}}\right)}^{2}+{\left({StDev}_{S1S2S3}\right)}^{2}}\right)}{753}}$$

#### Radiochemistry

**Scheme 1:**
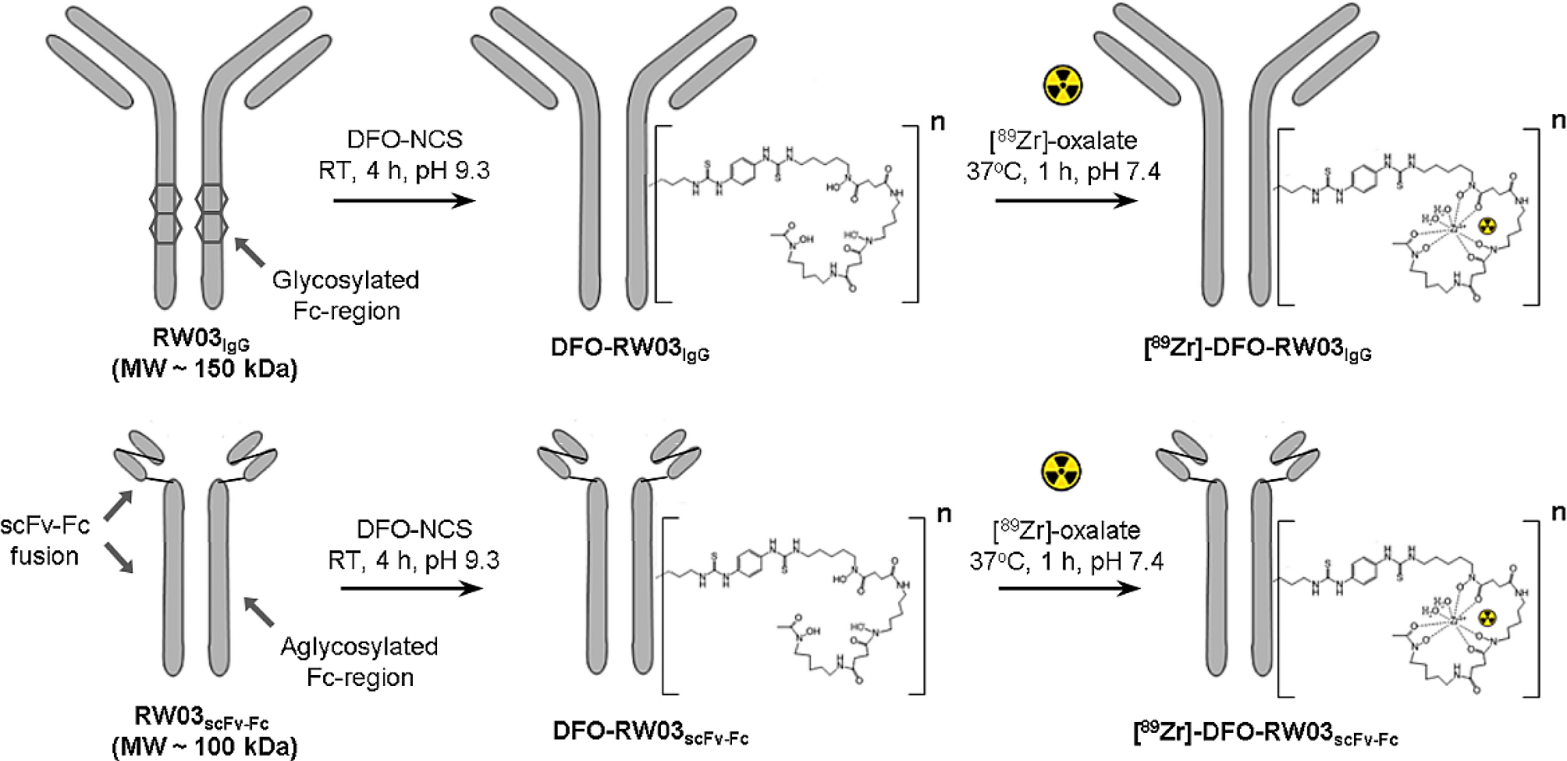
Conjugation and radiolabeling strategy to produce [^89^Zr]-DFO-RW03_IgG_ and [^89^Zr]-DFO-RW03_scFv − Fc_

Zirconium-89 radiolabeling was performed according to previously established procedures [20] (Scheme 1). In a typical labeling, 0.35 ± 0.15 mg conjugate was exchanged into HEPES buffer (1 M, pH 7.4) using a PD-10 column and concentrated to 1.25 mg/mL using centrifugal filtration. The [^89^Zr]-oxalate stock containing 185 MBq was diluted to 250 µL with oxalic acid (1 M). The [^89^Zr]-oxalate solution was slowly adjusted to pH 7 using a solution of Na_2_CO_3_ (1 M), monitored using pH strips. A 100 µL aliquot containing 74 MBq of the neutralized zirconium-89 solution was added to 0.35 ± 0.15 mg (280 ± 120 µL) of conjugate and incubated for 1 h at 37^o^C while shaking. The crude radiochemical yield was monitored by radio-iTLC at 0, 20, 40, and 60 min into the reaction. After 60 min, the reaction was quenched with 1 µl of EDTA (5 mM) for 10 min and purified using a PD-10 column equilibrated with ABST buffer. Fractions were characterized for radiochemical purity (RCP) by radio-iTLC, and fractions with > 97% RCP were pooled. The lower limit for specific activity was calculated using the activity of the final (pooled) product and the mass of IgG or scFv-Fc added to the reaction. After purification the product was filtered (0.22 µM) and activity losses to the filter were recorded.

#### Stability analysis

The radiochemical stability of [^89^Zr]-DFO-RW03_scFv − Fc_ was evaluated over 6 days in either saline or mouse serum. From the 37 MBq/mL stock solution of radioligand (in ABST), an aliquot (100 µL) was added to a 500 µL volume of either saline or mouse serum. Free zirconium-89 and soluble Zr^4+^ complex presence was evaluated by radio-iTLC up to 1-week post-synthesis using EDTA (0.1 M, pH 7) as the eluent.

#### Cell culture

HT-29 (HTB-38™) cells were purchased from ATCC and grown in McCoy’s 5 A medium (ATCC 30-2007™) supplemented with 10% FBS, penicillin (100 IU) and streptomycin (100 µg). Cells were incubated at 37^o^C in 95/5% air/CO_2_ humidified atmosphere. For subculturing (subculture ratio 10:1) and in vitro binding studies, media was removed and trypsin-EDTA (1 mL) (Thermo Fisher 15,050,057) was added for 5 min to liberate cells from the culture flask. Cells were passaged approximately every 3–4 d (80–90% confluency). All in vitro and in vivo experiments were performed at low (< 20) passage number.

#### Flow cytometry

To assess the effect of conjugation on antibody binding, HT-29 cells (3 × 10^6^ cells/mL) were incubated with native RW03_IgG_, native RW03_scFv − Fc_, or the DFO-RW03 conjugates to determine the half-maximal effective concentration (EC_50_) [14]. After incubation with the antibody or DFO-conjugate for 1 h on ice, cells were pelleted and washed to removed unbound antibody. Cells were then incubated with a secondary antibody (1:100 dilution) (Goat anti-Human IgG-FITC, BioRad, 204,002) for 30 min on ice. To stain dead cells, 7AAD (1:100) (Miltenyi 130-111-568) was added and incubated for at least 5 min. The median fluorescence intensity (MFI) for each measurement was plotted against antibody concentration and data was normalized to the highest MFI. The curves were fitted with a one-site binding sigmoidal curve using GraphPad Prism 8 to determine the EC_50_ values.

### Animal experiments

#### General

Animal experiments performed at McMaster University were approved by the Animal Research Ethics Board (AREB) at McMaster University. Animal experiments conducted with the Spatio-Temporal Targeting and Amplification of Radiation Response (STTARR) Innovation Centre (Toronto, Canada) were approved by the University Health Network (UHN). Animal experiments adhered to all applicable institutional and national guidelines, including guidelines of the Canadian Council on Animal Care and the Committee for Research and Ethical Issues of the International Association for the Study of Pain. Female BALB/c nu/nu mice (Charles River Laboratories, St Constant, QC) at 4–6 weeks of age were sterile housed and maintained at 24 °C with a 12 h light/dark cycle and were provided autoclaved food and water *ad libitum*. HT-29 xenografts were established in the hind limb by subcutaneous injection of 2 × 10^6^ cells formulated in 1:1 sterile DPBS (ThermoFisher 14,287,080)/Matrigel BD (Matrigel Matrix High Concentration, Phenol Red-Free, LDEV-Free, Corning 354,262). Radiotracer injections were performed 10 days post-tumor engraftment. All radiotracer injections were intravenous (i.v.) and administered through the lateral tail vein. Mice were humanely euthanized under anesthesia (5% isoflurane) by cervical dislocation. For biodistribution studies, blood, adipose tissue, bone, brain, heart, kidneys, large intestine and cecum (with contents), liver, lungs, pancreas, skeletal muscle, small intestine (with contents), spleen, stomach (with contents), bladder with urine and tail were collected at pre-determined timepoints, weighed, and counted in an automated gamma counter (Wallac Wizard 1470 gamma counter, PerkinElmer). Decay correction was used to normalize organ activity measurements to the time of dose preparation for data calculations with respect to the injected dose (reported as % injected dose per gram (%ID/g) and per organ (%ID/O)).

For PET imaging, probes [^89^Zr]-DFO-RW03_IgG_ (CA = 0.7 ± 0.1) and [^89^Zr]-DFO-RW03_IgG_ (CA = 3.0 ± 0.3) were imaged at STTARR using a Mediso nanoScan® SPECT/CT/PET system (Budapest, Hungary). PET images were acquired for 15 min and reconstructed using a voxel size of 0.4 mm, 4 iterations, and 4 ordered subsets. PET imaging of mice administered [^89^Zr]-DFO-RW03_scFv − Fc_ (CA = 2.9 ± 0.3) was performed at McMaster University using a LabPETII scanner (Imaging Research & Technology). Images were acquired for 20 min and images were reconstructed using a voxel size of 0.4 mm, 10 iterations, and 4 ordered subsets. Image processing and region-of-interest (ROI) analysis was performed using AMIDE [21].

#### Biodistribution experiments

The biodistribution of [^89^Zr]-DFO-RW03_IgG_ (CA = 3.0 ± 0.3) (0.14 ± 0.03 MBq, 1.0 ± 0.3 µg) in HT-29 xenograft mice was evaluated at 24, 48, and 96 h p.i. (*n* = 3 per timepoint). The biodistribution of [^89^Zr]-DFO-RW03_IgG_ (CA = 0.7 ± 0.1) (0.16 ± 0.02 MBq, 5.0 ± 0.7 µg) in HT-29 xenograft mice was evaluated at 24, 48, and 96 h p.i. (*n* = 3 per timepoint). To evaluate whether tumor uptake of [^89^Zr]-DFO-RW03_IgG_ (CA = 0.7 ± 0.1) was driven by antibody-antigen binding, the biodistribution [^89^Zr]-DFO-RW03_IgG_ (CA = 0.7 ± 0.1) (0.25 ± 0.01 MBq, 1.8 ± 0.1 µg) (non-blocked) was compared to [^89^Zr]-DFO-RW03 _IgG_ (CA = 0.7 ± 0.1) (0.33 ± 0.03 MBq, 2.5 ± 0.1 µg + 400 µg unlabeled RW03_IgG_) (blocked) in xenograft mice at 96 h p.i. (*n* = 3 per cohort per timepoint).

The biodistribution of [^89^Zr]-DFO-RW03_scFv − Fc_ (CA = 2.9 ± 0.3) (0.085 ± 0.02 MBq, 1.0 ± 0.2 µg) in HT-29 xenograft mice was evaluated at 24, 48, and 96 h p.i. (*n* = 3 per timepoint). To evaluate whether tumor uptake was driven by antibody-antigen binding, the biodistribution [^89^Zr]-DFO-RW03_scFv − Fc_ (CA = 2.9 ± 0.3) (0.09 ± 0.02 MBq, 1.0 ± 0.2 µg) (non-blocked) was compared to [^89^Zr]-DFO-RW03_scFv − Fc_ (CA = 2.9 ± 0.3) (0.09 ± 0.02 MBq, 1.0 ± 0.2 µg + 400 µg unlabeled RW03_IgG_) (blocked) in xenograft mice at 24 h p.i. (*n* = 3 per cohort per timepoint).

#### PET imaging

To evaluate the capability of each probe to image CD133-expressing tumors, HT-29 xenograft mice (*n* = 5 per group) were i.v. injected with either [^89^Zr]-DFO-RW03_IgG_ (CA = 0.7 ± 0.1) (1.8 ± 0.1 MBq, 14 ± 1 µg), [^89^Zr]-DFO-RW03_IgG_ (CA = 3.0 ± 0.3) (5.0 ± 0.2 MBq, 27 ± 1 µg), or [^89^Zr]-DFO-RW03_scFv − Fc_ (CA = 2.9 ± 0.3) (1.2 ± 0.1 MBq, 16 ± 2 µg) and imaged by PET after 24, 48, 96, and 168 h post injection.

To evaluate whether tumor uptake of [^89^Zr]-DFO-RW03_scFv − Fc_ (CA = 2.9 ± 0.3) was driven by antibody-antigen binding at PET imageable doses, mice were administered either [^89^Zr]-DFO-RW03_scFv − Fc_ (1.42 ± 0 0.07 MBq, 16.0 ± 0.2 ug) (non-blocking) or a co-injection of [^89^Zr]-DFO-RW03_scFv − Fc_ (1.40 ± 0.09 MBq, 16.0 ± 0.3 ug) and 0.5 mg RW03_IgG_ (blocking), and imaged using PET at 1 h, 24 h, 48 h, 72 h, 96 h, and 168 h post-injection (*n* = 5 per cohort). In each study, mice were euthanized, and organs were processed for biodistribution after the last imaging timepoint.

#### Statistical analysis

Unless otherwise noted, data is presented as the mean ± SEM. All tests for statistical significance were performed using GraphPad Prism 8. Asymmetrical confidence intervals (95% CI) were used to statistically describe results for in vitro cell binding assays. Student’s t-test was used to describe statistically significant differences (*p* < 0.05) in radioimmunoconjugate organ uptake between cohorts.

## Results

### Conjugate preparation

CD133-targeted PET imaging probes were synthesized using a fully human CD133 antibody, RW03_IgG_, or scFv-Fc fragment, RW03_scFv − Fc_, through conjugation with *p*-SCN-Bn-DFO (Scheme 1). MALDI-MS analysis showed that conjugation of RW03_IgG_ with a 5- or 30- fold molar excess of DFO-NCS produced conjugates with 0.7 ± 0.1 and 3.0 ± 0.3 DFO per antibody, respectively, and conjugation of RW03_scFv − Fc_ with a 10-fold molar excess of DFO-NCS produced a conjugate with 2.9 ± 0.3 DFO per scFv-Fc (Table S1-1). The mass of IgG or scFv-Fc recovered (% yield, Table S1-1) was 72 ± 5% for DFO-RW03_IgG_ (CA = 0.7 ± 0.1) and 80 ± 6% for DFO-RW03_scFv − Fc_ (CA = 2.9 ± 0.3), while only 33 ± 10% of DFO-RW03_IgG_ (CA = 3.0 ± 0.3) was recovered. Hereafter, DFO-RW03_scFv − Fc_ (C/A = 2.9 ± 0.3), DFO-RW03_IgG_ (C/A = 3.0 ± 0.3), and DFO-RW03_IgG_ (C/A = 0.7 ± 0.1) are shortened to DFO(2.9)-RW03_scFv − Fc_, DFO(3.0)-RW03_IgG_, and DFO(0.7)-RW03_IgG_, respectively. SEC-HPLC on the native RW03_IgG_ (Fig. S1-1b) and DFO(0.7)-RW03_IgG_ showed a smaller dimer peak (< 5% area) (Rt = 7.3 min) followed by a large monomeric peak (Rt = 8.2 min). The chromatogram for RW03_scFv − Fc_ (Fig. S1-1d) showed a substantial concentration (31% of total peak area) of scFv-Fc dimers and higher-order species (7% area) present in addition to the monomer scFv-Fc (60% area). Interestingly, these dimer and oligomeric species were not observed in the SEC-HPLC chromatogram for DFO(2.9)-RW03_scFv − Fc_ (Fig. S1-1e).

### Radiochemistry and stability studies

Radiolabeling was monitored by radio-iTLC until no further improvements in RCY were observed, which typically fell between 40 and 60 min with a crude RCY of 37 − 47% for [^89^Zr]-DFO(0.7)-RW03_IgG_, 95 − 99% for [^89^Zr]-DFO(3.0)-RW03_IgG_, and 74 – 98% for [^89^Zr]-DFO-RW03_scFv − Fc_ (Table S2-1, Fig. S2-1).

The radiochemical purity (RCP) of all products exceeded > 97% following SEC purification. Specific activities of 120 ± 30, 270 ± 90, and 200 ± 60 MBq/mg were achieved for [^89^Zr]-DFO(0.7)-RW03_IgG_, [^89^Zr]-DFO(3.0)-RW03_IgG_, and [^89^Zr]-DFO(2.9)-RW03_scFv − Fc_, respectively. The stability of [^89^Zr]-DFO(2.9)-RW03_scFv − Fc_ was monitored in both saline and mouse plasma (Fig. S2-2) through its radiochemical purity, measured by radio-iTLC, and showed a steady decrease in RCP from > 97% (t = 0 days) to < 70% (saline) and < 60% (plasma) at t = 6 days (Fig. S2-2).

### In vitro binding of DFO-RW03_scFv-Fc_ and [^89^Zr]-DFO-RW03_scFv-Fc_

Flow cytometry was used to compare the half-maximal effective concentration (EC_50_) of native RW03_IgG,_ DFO(0.7)-RW03_IgG_, DFO(3.0)-RW03_IgG_, native RW03_scFv − Fc_, and DFO(2.9)-RW03_scFv − Fc_ (Figure S3-1, Table S3-1). EC_50_ values (95% CI) for the native RW03_IgG_ and RW03_scFv − Fc_ were 0.6 to 1.6 nM and 1.5 to 3.3 nM, respectively. Unlabeled DFO(0.7)-RW03_IgG_, DFO(3.0)-RW03_IgG_, and DFO(2.9)-RW03_scFv − Fc_ had EC_50_ values of 0.6 to 1.1 nM, 0.3 to 1.9 nM, and 1.1 to 2.3 nM, respectively.

### Biodistribution in HT-29 xenograft mice

**Fig. 1:**
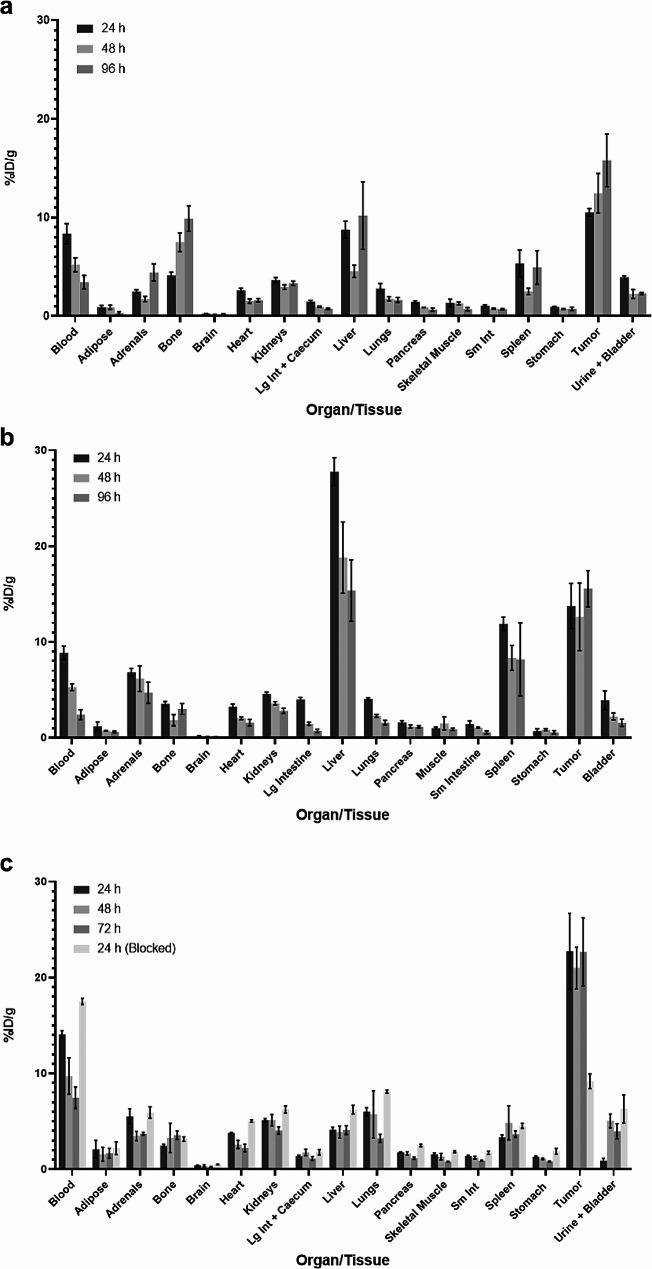
Biodistribution of [^89^Zr]-DFO(0.7)-RW03_IgG_, [^89^Zr]-DFO(3.0)-RW03_IgG_, [^89^Zr]-DFO(2.9)-RW03_scFv-Fc_ in HT-29 xenograft BALB/c nu/nu mice. (**a**) [^89^Zr]-DFO(0.7)-RW03_IgG_ (0.16 ± 0.02 MBq, 5 ± 0.7 ug) at 24, 48, 96 h p.i., (**b**) [^89^Zr]-DFO(3.0)-RW03_IgG_ (0.14 ± 0.03 MBq, 1 ± 0.3 µg) at 24, 48, 96 h p.i., (**c**) [^89^Zr]-DFO(2.9)-RW03_scFv-Fc_ (0.085 ± 0.02 MBq, 1 ± 0.2 µg) at 24, 48, and 72 h p.i. The blocked cohort received [^89^Zr]-DFO-RW03_scFv-Fc_ (0.09 ± 0.02 MBq, 1.0 ± 0.2 µg) and excess RW03_IgG_ (400 µg). Uptake is expressed as mean percentage injected dose per gram tissue (%ID/g) ± SEM.

Biodistribution experiments were conducted to establish tumor and off-target uptake of [^89^Zr]-DFO(0.7)-RW03_IgG_ (Fig. 1a), [^89^Zr]-DFO(3.0)-RW03_IgG_ (Fig. 1b), and [^89^Zr]-DFO(2.9)-RW03_scFv − Fc_ (Fig. 1c) (Tables S4-1 to S4-6).

**Fig. 2:**
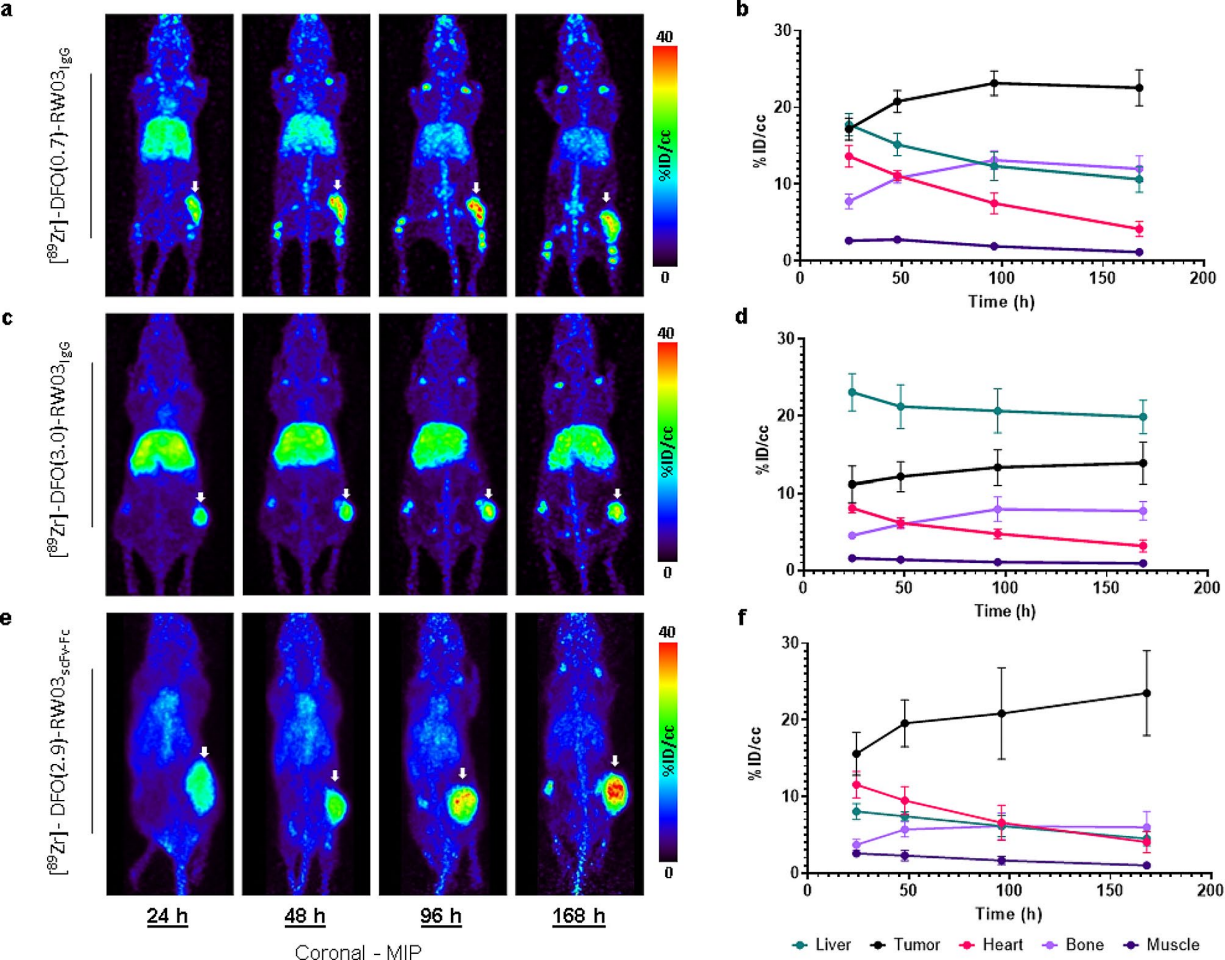
In vivo PET images (**a,c,e**) and corresponding ROI analysis (b, d, f) of [^89^Zr]-RW03 based radioligands: (**a & b**) [^89^Zr]-DFO(0.7)-RW03_IgG_ (1.27 ± 0.03 MBq, 10 ± 0.2 ug), (**c & d**) [^89^Zr]-DFO(3.0)-RW03_IgG_ (5.2 ± 0.4 MBq, 27 ± 2 µg), (**e & f**) [^89^Zr]-DFO(2.9)-RW03_scFv − Fc_ (1.2 ± 0.1 MBq, 16 ± 2 µg) in HT-29 xenograft BALB/c nu/nu mice. For PET images (**a, c, e**), the coronal plane is presented as a maximum intensity projection (MIP). White arrow = tumor. For ROI analysis (**b, d, f**) uptake is expressed as mean radioactivity per mL tissue volume (%ID/cc) ± SEM

The maximum uptake of [^89^Zr]-DFO(0.7)-RW03_IgG_ and [^89^Zr]-DFO(3.0)-RW03_IgG_ was similar in the blood (8 ± 1%ID/g and 8.8 ± 0.6%ID/g at 24 h, respectively) and in the tumor (16 ± 3%ID/g and 16 ± 2%ID/g at 96 h, respectively). Pronounced differences in maximum uptake of [^89^Zr]-DFO(0.7)-RW03_IgG_ and [^89^Zr]-DFO(3.0)-RW03_IgG_ were observed in the liver (8.7 ± 0.8%ID/g at and 27 ± 1%ID/g at 24 h, respectively) and the bone (9 ± 1%ID/g at and 3.0 ± 0.5%ID/g at 96 h, respectively). The biodistribution of [^89^Zr]-DFO(2.9)-RW03_scFv − Fc_ (Fig. 2) showed both higher tumor uptake and lower off-target organ uptake in contrast to the full IgG-based probes. The concentration of [^89^Zr]-DFO(2.9)-RW03_scFv − Fc_ in the blood halved between 24 h (14.1 ± 0.4%ID/g) and 72 h (7 ± 1%ID/g) and uptake in the liver was lower than blood uptake at all timepoints (liver: 4.1 ± 0.3, 3.8 ± 0.6, and 4.1 ± 0.5%ID/g at 24 h, 48 h, and 72 h, respectively). Bone uptake was lower than liver and blood uptake at all timepoints and measured 3.5 ± 0.1, 3 ± 1, and 3.7 ± 0.2%ID/g at 24 h, 48 h, and 72 h, respectively. Tumor uptake exceeded uptake in all off-target organs at all timepoints tested and measured 23 ± 4, 21 ± 3, and 23 ± 4%ID/g at 24 h, 48 h, and 72 h, respectively.

Antibody-antigen mediated tumor uptake of [^89^Zr]-DFO(2.9)-RW03_scFv − Fc_ was evaluated through co-injection of unlabeled RW03_IgG_ with [^89^Zr]-DFO(2.9)-RW03_scFv − Fc_ to block antigen in the tumor (Fig. 1c). Mice which received the blocking excess of RW03_IgG_ exhibited significantly reduced (*p* = 0.028) uptake of [^89^Zr]-DFO(2.9)-RW03_scFv − Fc_ in the tumor (9.2 ± 0.8%ID/g) relative to mice that received no block (23 ± 4%ID/g). Uptake in most off-target organs was not significantly different between the cohorts with exception of the blood which was significantly higher (*p* = 0.002) in the cohort that received excess RW03_IgG_ (17.5 ± 0.3 vs. 14.1 ± 0.4%ID/g). Supporting biodistribution tables for all studies are found in Supplementary 4.

### PET imaging studies

PET imaging studies were conducted to demonstrate the capacity for each probe for accumulate in and delineate CD133 expressing HT-29 tumor xenografts (Fig. 2, Tables S4-7 to S4-12). Images show each tracer accumulating in the tumor as early as 24 h after injection and increasing until a maximum is reached at 96 or 168 h. Uptake of [^89^Zr]-DFO(0.7)-RW03_IgG_ in the tumor reached 17.2 ± 0.7%ID/cc after 24 h, reaching a maximum of 23.2 ± 0.7%ID/cc after 96 h with a maximum tumor/liver ratio of 2.2:1 and tumor/heart ratio of 5:1 after 168 h. For [^89^Zr]-DFO(3.0)-RW03_IgG_, uptake in the tumor reached 11.2 ± 1.0%ID/cc after 24 h, reaching a maximum of 13.4 ± 1.2%ID/cc after 168 h with a maximum tumor/liver ratio of 0.73:1 and tumor/heart ratio of 4:1 after 168 h. In contrast to the IgGs based probes, tumor uptake of [^89^Zr]-DFO(2.9)-RW03_scFc − Fc_ reached 15.6 ± 1.4%ID/cc after 24 h, reaching a maximum of 23.5 ± 3%ID/cc after 168 h with a maximum tumor/liver ratio of 5.2:1 and tumor/heart ratio of 5.8:1 after 168 h. Higher joint uptake over the duration of the study was seen for [^89^Zr]-DFO(0.7)-RW03_IgG_ in contrast with [^89^Zr]-DFO(3.0)-RW03_IgG_ and [^89^Zr]-DFO(2.9)-RW03_scFv − Fc_, where at 168 h joint uptake for each probe reached 12.0 ± 0.8%ID/cc, 7.7 ± 0.6%ID/cc, and 6.0 ± 1.0%ID/cc, respectively.

**Fig. 3:**
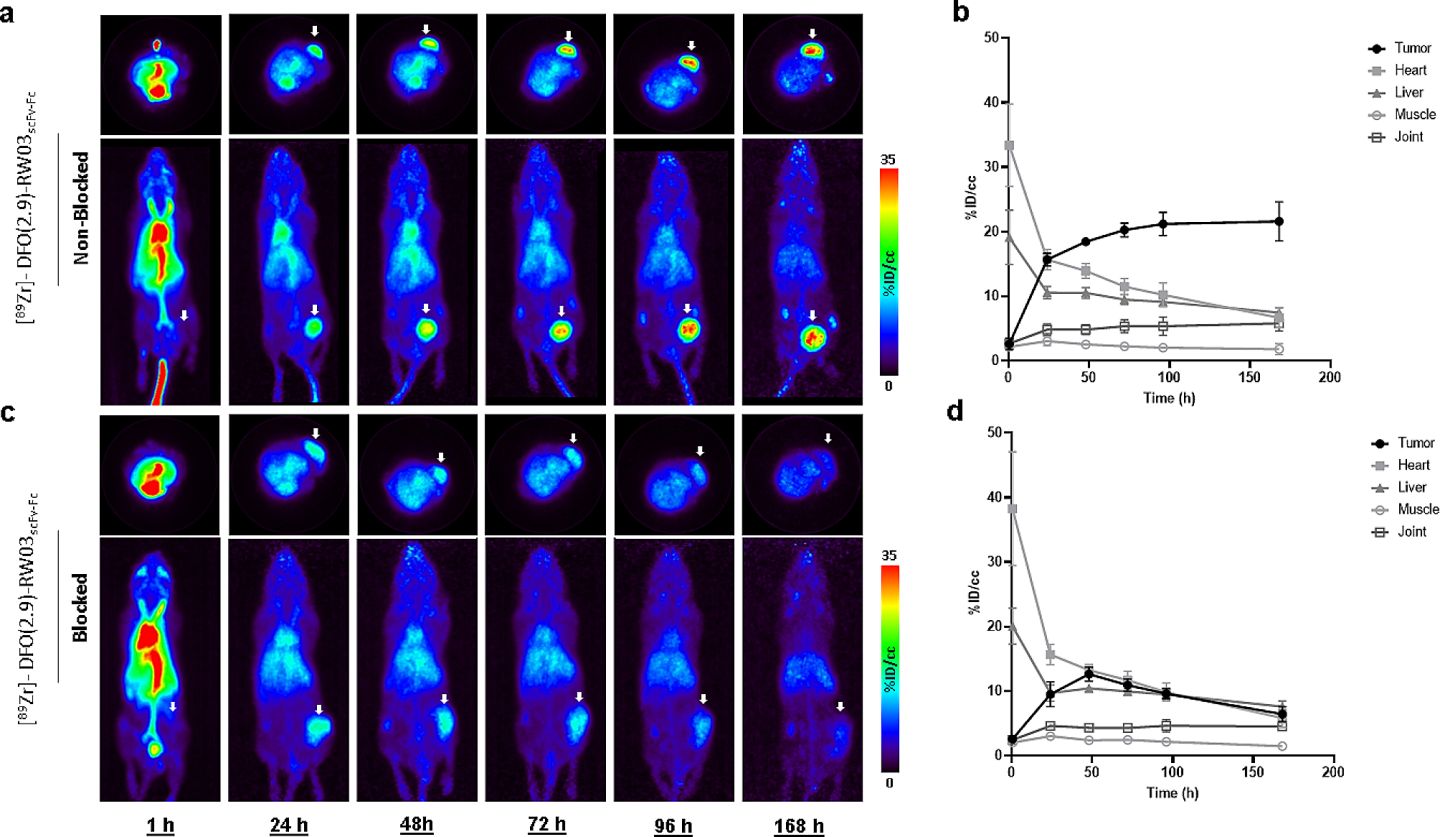
[^89^Zr]-DFO-RW03_scFv-Fc_ ImmunoPET imaging CD133 expressing tumors. [^89^Zr]-DFO(2.9)-RW03_scFv-Fc_ PET imaging study (**a & c**) and corresponding ROI analysis (**b & d**). Mice received (**a & b**) [^89^Zr]-DFO(2.9)-RW03_scFv-Fc_ (1.42 ± 0.07 MBq, 16.0 ± 0.2 ug) or (**c & d**) [^89^Zr]-DFO(2.9)-RW03_scFv-Fc_ (1.40 ± 0.09 MBq, 16.0 ± 0.3 ug) co-injected with RW03_IgG_ (0.5 mg). For PET images, the transverse and coronal planes are presented as maximum intensity projections (MIP). White arrow = tumor. For ROI analysis (**b, d**) uptake is expressed as mean radioactivity per mL tissue volume (%ID/cc) ± SEM

To further demonstrate the capability for [^89^Zr]-DFO(2.9)-RW03_scFv − Fc_to accumulate in CD133 expressing tumors, a PET-imaging study was conducted where mice were administered [^89^Zr]-DFO(2.9)-RW03_scFv − Fc_in the presence and absence of unlabeled RW03 (Fig. 3, Tables S4-3 & S4-4). Images of both cohorts at 1 h p.i. showed high uptake of [^89^Zr]-DFO(2.9)-RW03_scFv − Fc_ in blood pool (heart) (no RW03_IgG_: 33 ± 5%ID/cc, with RW03_IgG_: 38 ± 4%ID/cc) and low tumor uptake (< 3%ID/cc). The tumor uptake of [^89^Zr]-DFO(2.9)-RW03_scFv − Fc_ increased over the duration of the study, with tumor uptake at 72 h (20.3 ± 0.5%ID/cc) in good agreement with tumor uptake observed at 72 h in the biodistribution study (23 ± 4%ID/g) (see Fig. 1). As early as 24 h p.i. the tumor-to-heart ratio equalized in the non-blocked cohort and at this timepoint a significant reduction (*p* = 0.006) in tumor uptake was observed in the blocked cohort (10 ± 1%ID/cc) relative to the non-blocked cohort (15.6 ± 0.5%ID/cc). The tumor-to-blood ratio and tumor-to-liver ratio increased in the non-blocked cohort over the duration of the study, and both reached a maximum of 3:1 at 168 h p.i. The tumor-to-blood ratio and tumor-to-liver ratio in the blocked cohort equalized at 72 h and 24 h p.i., respectively, and both ratios stayed at 1:1 over the duration over the study (168 h p.i.). Uptake in the knee joint contralateral to the tumor was highest at the last imaging timepoint (168 h p.i.) in both cohorts (5.8 ± 0.6%ID/cc and 4.5 ± 0.2%ID/cc for the non-blocked and blocked cohorts, respectively). The ROI analysis at 168 h p.i. is in good agreement with ex vivo biodistribution performed on mice from the imaging study, which found tumor uptake of 18.2 ± 0.6%ID/g and 5 ± 1%ID/g in the non-blocked and blocked cohorts at 168 h, respectively (Fig. S4-1, Tables S4-5 & S4-6).

## Discussion

Cancer stem cell-targeted immunoPET probes could aid in the development and utilization of CSC-targeted therapies by identifying CSC-rich tumors for patient selection or by detecting residual CSCs in patients following treatment [22]. CD133 antigen is selective for CSCs in a wide variety of tumors [3–7], which has driven development of CD133-targeted therapies aimed to eliminate CSCs for improved treatment outcomes [8–10]. To aid in clinical translation of these therapies, previously reported molecular imaging tools have utilized CD133-targeted humanized or murine monoclonal antibodies, which while demonstrating promising preclinical data, have faced barriers to clinical translation due to immunogenicity concerns [14, 23]. Here, we report the synthesis of CD133-targeting immunoPET probes, [^89^Zr]-DFO(0.7)-RW03_IgG_, [^89^Zr]-DFO(3.0)-RW03_IgG_, and [^89^Zr]-DFO(2.9)-RW03_scFv − Fc_ using a fully human IgG and scFv-Fc fragment, and demonstrate the capability of these probes to visualize CD133-expressing tumors in mice through PET imaging.

Intact IgGs and scFv-Fc antibody fragments demonstrate similar pharmacokinetics owing to the fragment crystallizable (Fc)-region, which prolongs blood circulation through saturable binding with Fc-receptors present in the liver and other tissues [24]. For both IgGs and scFv-Fcs, the long half-life (3.3 days) of zirconium-89 and ability to radiolabel proteins under non-denaturing conditions make it an attractive isotope for immunoPET probes. We found that conjugation of RW03_scFv − Fc_ with DFO did not reduce the CD133-binding affinity of the conjugates relative to native RW03_IgG_ or RW03_scFv − Fc_ for up to 3 DFO per antibody, in-line with previously reported results [25].

Previous reports have demonstrated that a high chelator-to-antibody ratio (C/A) can be detrimental to tumor uptake and can drive high liver uptake which could prohibit imaging of tumors in the vicinity of the liver [25]. In biodistribution studies, the tumor uptake of [^89^Zr]-DFO(2.9)-RW03_scFv − Fc_ exceeded liver uptake at all timepoints (tumor/liver ratios of 5.6 : 1, 5.4 : 1, and 5.6 : 1 at 24, 48, and 96 h, respectively) indicating that the chelator-to-antibody ratio did not lead to unmanageable liver uptake for this probe. In contrast, [^89^Zr]-DFO(3.0)-RW03_IgG_ showed higher liver uptake than tumor uptake at 24 h (liver = 28 ± 1%ID/g, tumor = 14 ± 2%ID/g) and 48 h (liver = 18 ± 4%ID/g, tumor = 13 ± 4%ID/g) post-injection. In line with these results, [^89^Zr]-DFO(2.9)-RW03_scFv − Fc_ readily delineated tumor from liver in PET imaging studies at 24 h p.i. (tumor/liver ratio = 1.9) out to 168 h p.i. (tumor/liver ratio = 5.2), whereas the tumor/liver ratio for [^89^Zr]-DFO(3.0)-RW03_IgG_ was < 1 at each timepoint throughout the 168 h imaging study. While the tumor/liver ratio using [^89^Zr]-DFO(0.7)-RW03_IgG_ was improved over [^89^Zr]-DFO(3.0)-RW03_IgG_, it remained lower at each timepoint than [^89^Zr]-DFO(2.9)-RW03_scFv − Fc_. The superior biodistribution and imaging results of [^89^Zr]-DFO(2.9)-RW03_scFv − Fc_ highlight this probe as the most effective synthesized here for CD133 tumor imaging.

Previous reports on CD133-imaging probes include a zirconium-89 labeled commercially available humanized anti-CD133 antibody, [^89^Zr]-DFO-AC133.1, which was administered to CD133 expressing human colorectal cancer xenograft BALB/c nu/nu mice at a dose of 1.85 MBq (30 µg). This radioimmunoconjugate demonstrated a maximum of 13%ID/g tumor uptake and minimum of 4%ID/g liver uptake (tumor/liver ratio = 3.25) at 6 days post-injection [13], which is exceeded by the tumor/liver ratio of 5.2 provided by [^89^Zr]-DFO(2.9)-RW03_scFv − Fc_ at 168 h p.i. Other reports on antibody-based CD133 immunoPET utilize AC133.1 and other anti-CD133 targeted antibodies to show uptake and imaging of orthotopic PDX glioblastoma and PDX lung cancer mouse models, with tumor-to-off target uptake exceeding that provided by [^89^Zr]-DFO(2.9)-RW03_scFv − Fc_ [14, 16]. The HT-29 tumor xenograft model robustly expresses CD133 [26, 27] and facilitated initial investigations into the capability of CD133-imaging using [^89^Zr]-DFO-RW03_scFv − Fc_. Patient-derived orthotopic tumor models with clinically representative CD133 populations represent the next step for evaluation of RW03-based PET probes.

Previous reports have demonstrated decreased tumor uptake at higher injected antibody doses, with one report finding that increasing the injected antibody dose 10-fold (from 1 to 10 µg) led to about a 50% decrease in tumor uptake (from ∼ 35 to 17%ID/g at 72 h p.i. for 1 and 10 µg injected doses, respectively) [28]. PET imaging studies on [^89^Zr]-DFO-RW03_scFv − Fc_ used a 16-fold higher antibody dose than that used in biodistribution studies (16.0 ± 0.2 µg vs. 1.0 ± 0.2 µg for PET and biodistribution studies, respectively) and tumor uptake between the two studies did not significantly differ (e.g. 20.3 ± 0.5%ID/cc and 23 ± 4%ID/g for PET and biodistribution at 24 h p.i., respectively). For clinical studies it may be necessary to optimize the amount of cold antibody used. For example, in the ZIPHIR Trial (NCT01565200) patients received 37 MBq of [^89^Zr]-labeled trastuzumab and 50 mg cold trastuzumab for a final effective specific activity of 0.74 MBq/mg. The high effective SA achieved here for [^89^Zr]-DFO-RW03_scFv − Fc_ (220 MBq/mg) is promising for imaging the rare CSC population in tumors and provides a large window for titrating the amount of cold RW03_IgG_ that can be co-administered to reduce off-target uptake in a clinical setting.

### Summary and conclusions

[^89^Zr]-DFO-RW03_scFv − Fc_, a CD133 targeting immunoPET probe based on a fully human monoclonal antibody was developed and its ability to target CD133-expressing tumors demonstrated through biodistribution and PET imaging studies. These showed favorable tumor uptake and low off-target uptake, including in the liver and bone. [^89^Zr]-DFO-RW03_scFv − Fc_ is a promising immunoPET tracer as a companion diagnostic and warrants further evaluation in PDX tumor models that better represent CSC populations in patient tumors.

## Electronic supplementary material

Below is the link to the electronic supplementary material.

Supplementary Material 1

Supplementary Material 2

## Data Availability

All data generated or analyzed during this study are included in this published article [and its supplementary information files].

## Abbreviations

7AAD: 7-Aminoactinomycin D
ACS: American Chemical Society
BSA: Bovine Serum Albumin
CD133: Cluster of Differentiation 133
CI: Confidence Interval
CSC: Cancer Stem Cell
DF: Dilution Factor
DFO-NCS: Para-isothiocyanatobenzyl-deferoxamine
EC50: Half-Maximal Effective Concentration
FBS: Fetal Bovine Serum
FITC: Fluorescein Isothiocyanate
IRF: Immunoreactive Fraction
iTLC-SG: Instant Thin-Layer Chromatography on Silica Gel
MALDI-MS: Matrix-Assisted Laser Desorption/Ionization Mass Spectrometry
MFI: Median Fluorescence Intensity
MW: Molecular Weight
PET: Positron Emission Tomography
RCP: Radiochemical Purity
RCY: Radiochemical Yield
ROI: Region of Interest
RT: Room Temperature
SEC-HPLC: Size Exclusion Chromatography - High-Performance Liquid Chromatography
SEM: Standard Error of the Mean
TOF: Time of Flight
UV: Ultraviolet
