# Correction: ^89^Zr-labeled ImmunoPET targeting the cancer stem cell antigen CD133 using fully-human antibody constructs

**DOI:** 10.1186/s13550-025-01331-6

**Published:** 2025-11-04

**Authors:** Kevin Wyszatko, Melissa Chassé, Nancy Janzen, Luis Rafael Silva, Luke Kwon, Teesha Komal, Manuela Ventura, Chitra Venugopal, Sheila K. Singh, John F. Valliant, Saman Sadeghi

**Affiliations:** 1 Department of Chemistry and Chemical Biology, McMaster University, Hamilton, ON Canada; 2 Institute of Medical Science, Temerty Faculty of Medicine, University of Toronto, Toronto, ON Canada; 3 Spatio-Temporal Targeting and Amplification of Radiation Response Innovation Centre (STTARR), University Health Network, Toronto, ON Canada; 4 Centre for Discovery in Cancer Research, McMaster University, Hamilton, ON Canada; 5 Department of Surgery, McMaster University, Hamilton, ON Canada

**Correction: EJNMMI Research (2024) 14:29**

 10.1186/s13550-024-01091-9

Following publication of the original article, the author Melissa Chassé - who, owing to a miscommunication, had been omitted from the author list in error - has been added to the authorship of the article. Please refer to the current version of the author list. The authors thank you for reading this erratum and apologize for any inconvenience caused.

