# Supplementary material for: 89Zr-labeled ImmunoPET targeting the cancer stem cell antigen CD133 using fully-human antibody constructs
Source: EJNMMI Res. 2024 Mar 18;14:29. doi: 10.1186/s13550-024-01091-9 (PMC10948676; doi:10.1186/s13550-024-01091-9)
Supplement: Supplementary file 2 — Supplementary Material 2 [file 13550_2024_1091_MOESM2_ESM.docx]

**Supplementary Information**

**Title:**

**^89^Zr-labeled ImmunoPET Targeting the Cancer Stem Cell Antigen CD133 using Fully-Human Antibody Constructs**

**Authors:**

Kevin Wyszatko^†^, Nancy Janzen^†^, Luis Rafael Silva^†^, Luke Kwon^§^, Teesha Komal^§^, Manuela Ventura^§^, Chitra Venugopal^||^, Sheila Singh^||,#^, John F. Valliant^†^, Saman Sadeghi^†^*

**Affiliations**

^†^ Department of Chemistry and Chemical Biology, McMaster University, Hamilton, Ontario, Canada

^§^ Spatio-Temporal Targeting and Amplification of Radiation Response (STTARR) Innovation Centre, University Health Network, Toronto, ON, Canada

^||^Centre for Discovery in Cancer Research, McMaster University, Hamilton, Ontario, Canada;

^#^Department of Surgery, McMaster University, Hamilton, Ontario, Canada

*Corresponding author: Saman Sadeghi, sadegs10@mcmaster.ca, Department of Chemistry and Chemical Biology, McMaster University, Hamilton, Ontario, Canada

**Supplementary 1. Conugation and MALDI-MS Charaterization**

**Description for Table S1-1**

To characterize the number of DFO conjugated per RW03_IgG_ or RW03_scFv-Fc_, Matrix-Assisted Laser Desorption Ionization Mass Spectrometry (MALDI-MS) was performed using a Bruker UltrafleXtreme TOF/TOF in positive mode to determine the molecule weight of the parent antibody and conjugate. Samples (triplicate) were formulated at concentration of 1 mg/mL in phosphate buffer (0.02 M, pH 7.4) and then diluted 1x with a saturated solution of sinapinic acid in TA30 solvent (70 (v/v) acetonitrile:0.1% TFA in water solvent). From this mixture 1 µL was spotted on a steel target and left to dry before laser ablation. A solution of bovine serum albumen (BSA) was prepared and used as an external standard.

**Table S1-1.** MALDI-MS characterization used for C/A determination of DFO-RW03_IgG_ or DFO-RW03_scFv-Fc_ conjugates. Calculation of C/A was performed using molecular weight of 752.95 g/mol per addition of 1 molecule *p*-SCN-Bn-DFO.

| Sample | Molar Excess of  NCS*-*DFO in reaction | m/z (n = 3) | | m/z Increase | | C/A (n = 3) | | Mass yield (%) |
| --- | --- | --- | --- | --- | --- | --- | --- | --- |
|  |  | Mean | SD | Mean | SEM | Mean | SEM |  |
| RW03_IgG_ |  | 150040 | 80 |  |  |  |  |  |
| DFO-RW03_IgG_ | 5 | 150600 | 100 | 560 | 80 | 0.7 | 0.1 | 72 ± 5 |
| DFO-RW03_IgG_ | 30 | 152300 | 200 | 2260 | 220 | 3.0 | 0.3 | 33 ± 10 |
| RW03_scFv-Fc_ |  | 105600 | 100 |  |  |  |  |  |
| DFO-RW03_scFv-Fc_ | 10 | 107800 | 200 | 2200 | 200 | 2.9 | 0.3 | 80 ± 6 |

**Description for Figure S1-1**

SEC-HPLC characterization of a commercial isotype IgG, the native RW03_IgG_, the native scFv-Fc scaffold, and conjugates were performed on Waters 1525 Binary HPLC using a Yarra 3um SEC 3000 (300 × 7.8 mm) with a mobile phase of phosphate buffer (0.02 M, pH 6.7). Samples were analyzed using Bioscan 2489 Waters 2489 UV/Vis (λ = 280 nm). Spectra were recorded and processed on Empower 2 software (Waters).

HPLC chromatograms are shown in Fig. S1-1. In Fig. S1-1 (a) an isotype IgG is presented to demonstrate the resolution afforded by the column. It is worth noting that we were initially uncertain how the relatively high proportion of scFv-Fc dimer in solution (20 - 30 %) (Fig. S1-1 d) would impact immunoPET probes made from this scaffold. As seen in the chromatogram for DFO-RW03_scFv-Fc_ (Fig. S1-1e), conjugation with DFO-NCS to generate DFO-RW03_scFv-Fc_ (C/A = 2.9) reduced the propensity for this scaffold to multimerize.


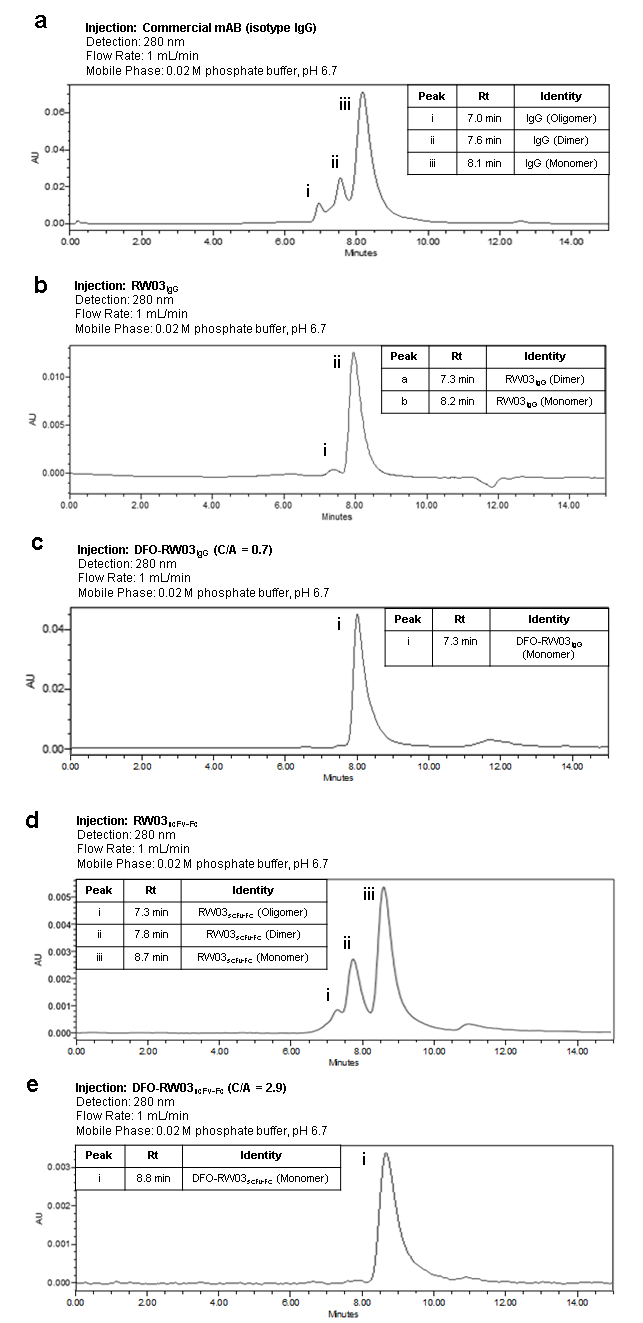


**Figure S1-1.** SEC-HPLC chromatograms for a) an isotype control IgG (150 µg), b) native RW03_IgG_ (20 µg)_,_ c) DFO-RW03_IgG_ (CA = 0.7 ± 0.1) (150 µg), d) native RW03_scFv-Fc_ (10 µg), e) DFO-RW03_scFv-Fc_ (CA = 2.9 ± 0.3) (7.5 µg). In d) multiple peaks are observed for the native RW03_scFv-Fc_, which reduced to a single peak in e) after conjugation with DFO-NCS.

**Online Resource 2. Radiochemistry**

**Description for Table S2-1 and Figure S2-1, 2-2**

Table S2-1 and Figures S2-1 to S2-2 used radio-iTLC to monitor [^89^Zr]-DFO-RW03_IgG_ (C/A = 0.7, 3.0) [^89^Zr]-DFO-RW03_scFv-Fc_ (C/A = 2.9) radiolabeling reaction progress and radiochemical purity. Radio-iTLCs were performed using iTLC-SG glass microfiber chromatography paper (SGI0001, Agilent Technologies) plates with sodium citrate buffer (0.1 M, pH 5) as the eluent. Radio-iTLCs were analyzed using a Bioscan AR-2000 Imaging Scanner.

**Table S2-1.** Reaction progress and isolated RCY for zirconium-89 radiolabeling of DFO-RW03_IgG/scFv-Fc_ conjugates. Isolated RCY represents the activity collected after PD-10 column purification and sterile filtration relative to the activity added to the reaction.

|  | [^89^Zr]-DFO-RW03_IgG_ (C/A = 0.7) | | [^89^Zr]-DFO-RW03_IgG_ (C/A = 3.0) | | [^89^Zr]-DFO-RW03_scFv-Fc_ (C/A = 2.9) | |
| --- | --- | --- | --- | --- | --- | --- |
| Time (min) | RCY (%) | SD |  |  | RCY (%) | SD |
| 0 | 9 | 8 | 42 | 11 | 29 | 21 |
| 20 | 15 | 10 | 89 | 7 | 78 | 18 |
| 40 | 32 | 8 | 93 | 6 | 84 | 14 |
| 60 | 42 | 5 | 97 | 2 | 86 | 12 |
| Isolated | 30 | 1 | 80 | 3 | 39 | 13 |


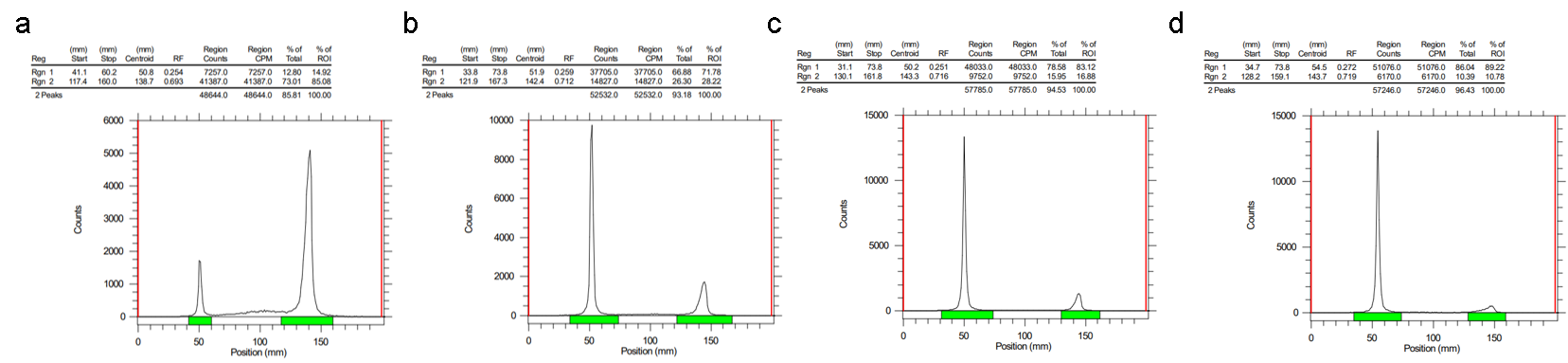


**Figure S2-1.** Representative radio-iTLC traces for zirconium-89 radiolabeling of DFO-RW03_scFc-Fc_ (C/A = 2.9 ± 0.3). Sampling peformed at a) t= 0 min, b) t = 20 min, c) t = 40 min, d) t = 60 min.


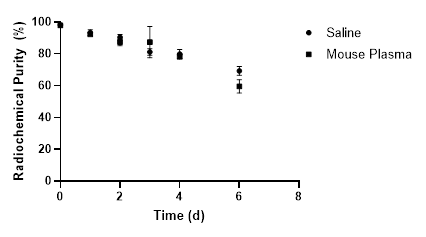


**Figure S2-2.** Radio-iTLC determination of [^89^Zr]-DFO-RW03_scFv-Fc_ (C/A = 2.9 ± 0.3) stability in saline and mouse serum.

**Supplementary 3. In Vitro Characterization**

**Description for Figure S3-1 and Table S3-1**

Flow cytometry was used to measure changes in the EC_50_ of native RW03_IgG_ and RW03_scFv-Fc_ after conjugation with DFO-NCS (Fig. S3-1). The EC_50_ values were taken as the half-maximal concentration required to induce a decrease in MFI which occurred when ligand concentrations > 10 nM (Table S3-1).


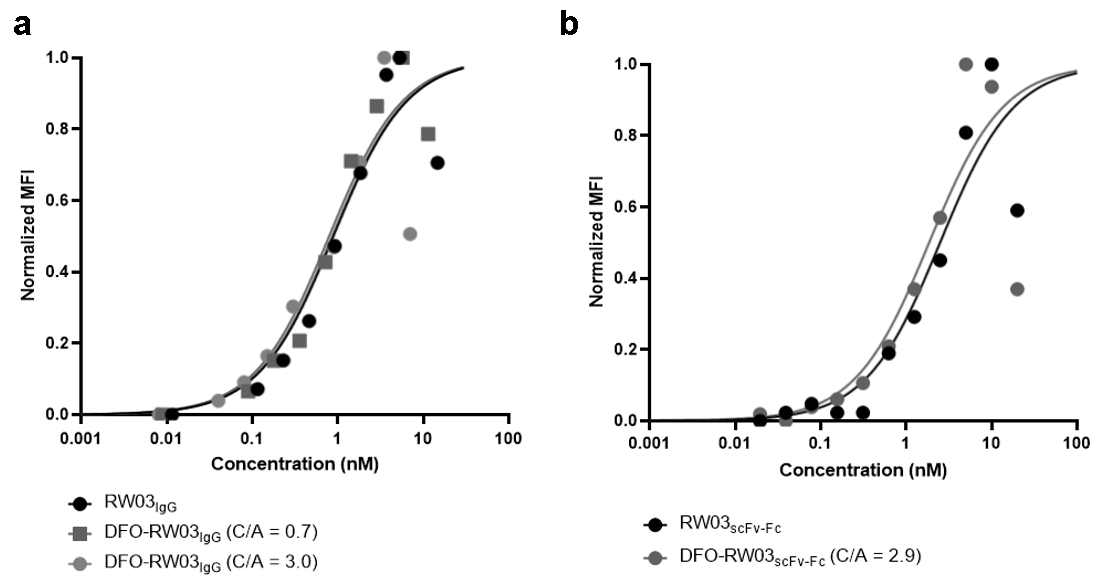


**Figure S3-1.** Flow cytometry saturation binding of a) RW03_IgG_, DFO-RW03_IgG_ (C/A = 0.7), DFO-RW03_IgG_ (C/A = 3.0) and b) RW03_scFv-Fc_ and DFO-RW03_scFv-Fc_ (C/A =2.9). Data presented as the normalized mean fluorescence intensity (MFI).

**Table S3-1.** EC_50_ values for RW03_IgG_, RW03_scFv-Fc_ and conjugates. The data was normalized and concentrations > 10 nM were excluded from the fit.

|  | |  | |  |  |  |  |
| --- | --- | --- | --- | --- | --- | --- | --- |
|  | RW03_IgG_ | | DFO-RW03_IgG_  (C/A = 0.7) | | DFO-RW03_IgG_  (C/A = 3.0) | RW03_scFv-Fc_ | DFO-RW03_scFv-Fc_ (C/A = 2.9) |
| Best-fit values |  | |  | |  |  |  |
| EC50 | 0.89 nM | | 0.79 nM | | 0.85 nM | 2.2 nM | 1.6 nM |
| 95% CI |  | |  | |  |  |  |
| EC50 | 0.6 to 1.3 nM | | 0.6 to 1.1 nM | | 0.3 to 1.9 nM | 1.5 to 3.3 nM | 1.1 to 2.3 nM |
| Goodness of Fit |  | |  | |  |  |  |
| R squared | 0.95 | | 0.96 | | 0.79 | 0.94 | 0.95 |

**Supplementary 4. Additional Biodistribution and Imaging Data**

**Description for Tables S4-1 to S4-6**

Biodistribution tables for Fig 1. & Fig. 2.

**Table S4-1.** Ex vivo biodistribution of [^89^Zr]-DFO-RW03_IgG_ (C/A = 0.7) (0.16 ± 0.02 MBq, 5 ± 0.7 µg) in HT-29 xenograft BALB/c nu/nu mice. Uptake is expressed as mean percentage injected dose per gram tissue (%ID/g).

**Table S4-2.** Ex vivo biodistribution of [^89^Zr]-DFO-RW03_IgG_ (C/A = 0.7) (0.16 ± 0.02 MBq, 5 ± 0.7 µg) in HT-29 xenograft BALB/c nu/nu mice. Uptake is expressed as mean percentage injected dose per organ (%ID/O).

**Table S4-3.** Ex vivo biodistribution of [^89^Zr]-DFO-RW03_IgG_ (C/A = 3.0) (0.14 ± 0.03 MBq, 1 ± 0.3µg) in HT-29 xenograft BALB/c nu/nu mice. Uptake is expressed as mean percentage injected dose per gram tissue (%ID/g).

**Table S4-4.** Ex vivo biodistribution of [^89^Zr]-DFO-RW03_IgG_ (C/A = 3.0) (0.14 ± 0.03 MBq, 1 ± 0.3µg) in HT-29 xenograft BALB/c nu/nu mice. Uptake is expressed as mean percentage injected dose per organ (%ID/O).

**Table S4-5.** Ex vivo biodistribution of [^89^Zr]-DFO-RW03_scFv-Fc_ (0.09 ± 0.02 MBq, 1*.*2 ± 0.2 µg) in HT-29 xenograft BALB/c nu/nu mice. The blocked cohort received [^89^Zr]-DFO-RW03_scFv-Fc_ (0.09 ± 0.02 MBq, 1 ± 0.2 µg) and excess RW03_IgG_ (400 µg). Uptake is expressed as mean percentage injected dose per gram tissue (%ID/g).

**Table S4-6.** Ex vivo biodistribution of [^89^Zr]-DFO-RW03_scFv-Fv_ (0.09 ± 0.02 MBq, 1.0 ± 0.2 µg) in HT-29 xenograft BALB/c nu/nu mice. The blocked cohort received [^89^Zr]-DFO-RW03_scFv-Fc_ (0.09 ± 0.02 MBq, 1 ± 0.2 µg) and excess RW03_IgG_ (400 µg). Uptake is expressed as mean percentage injected dose per organ (%ID/O).

**Description for Table S4-7 to S4-11**

ROI analysis tables and ex vivo biodistribution tables for Fig. 2.

**Table S4-7.** PET image ROI table. In vivo PET image ROI analysis of [^89^Zr]-DFO-RW03_IgG_ (C/A = 0.7) (1.27 ± 0.03 MBq, 10 ± 0.2 ug) in HT-29 xenograft BALB/c nu/nu mice. Uptake is expressed as mean radioactivity per mL tissue volume (%ID/cc). Data expressed as mean ± SEM.

**Table S4-8.** PET image ROI table. In vivo PET image ROI analysis of [^89^Zr]-DFO-RW03_IgG_ (C/A = 3.0) (5.2 ± 0.4 MBq, 27 ± 2 µg) in HT-29 xenograft BALB/c nu/nu mice. Uptake is expressed as mean radioactivity per mL tissue volume (%ID/cc). Data expressed as mean ± SEM.

**Table S4-9.** PET image ROI table. In Vivo PET image ROI analysis of [^89^Zr]-DFO-RW03_scFv-Fc_ (C/A=2.8) (1.2±0.1 MBq, 16±2 µg) in HT-29 xenograft BALB/c nu/nu mice. Uptake is expressed as mean radioactivity per mL tissue volume (%ID/cc). Data expressed as mean±SEM.

**Table S4-10.** Ex vivo biodistribution tables from PET imaging study of [^89^Zr]-DFO-RW03_IgG_ (C/A = 0.7) (1.27 ± 0.03 MBq, 10 ± 0.2 ug), [^89^Zr]-DFO-RW03_IgG_ (C/A = 3.0) (5.2 ± 0.4 MBq, 27 ± 2 µg), and [^89^Zr]-DFO-RW03_scFv-Fc_ (C/A=2.8) (1.2±0.1 MBq, 16±2 µg) in HT-29 xenograft BALB/c nu/nu mice at 168 h. Uptake is expressed as mean percentage injected dose per gram tissue (%ID/g).

**Table S4-11.** Ex vivo biodistribution tables from PET imaging study of [^89^Zr]-DFO-RW03_IgG_ (C/A = 0.7) (1.27 ± 0.03 MBq, 10 ± 0.2 ug), [^89^Zr]-DFO-RW03_IgG_ (C/A = 3.0) (5.2 ± 0.4 MBq, 27 ± 2 µg), and [^89^Zr]-DFO-RW03_scFv-Fc_ (C/A=2.8) (1.2±0.1 MBq, 16±2 µg) in HT-29 xenograft BALB/c nu/nu mice at 168 h. Uptake is expressed as mean percentage injected dose per gram tissue (%ID/O).

**Description for Table S4-12 to S4-15**

ROI analysis tables and ex vivo biodistribution tables for Fig. 3.

**Table S4-12.** ROI analysis of [^89^Zr]-DFO-RW03_scFv-Fc_ (1.42 ± 0.07 MBq, 16.0 ± 0.2 µg) (non-blocked). Uptake is expressed as mean percentage injected dose per unit volume (%ID/cc).

|  | 0.5 h | | 24 h | | 48 h | | 72 h | | 96 h | | 168 h | |
| --- | --- | --- | --- | --- | --- | --- | --- | --- | --- | --- | --- | --- |
|  | Mean | SEM | Mean | SEM | Mean | SEM | Mean | SEM | Mean | SEM | Mean | SEM |
| Tumor | 2.6 | 0.6 | 15.6 | 0.5 | 18.4 | 0.3 | 20.3 | 0.5 | 21.2 | 0.9 | 22 | 2 |
| Heart | 33 | 5 | 15.7 | 0.8 | 13.9 | 0.6 | 11.5 | 0.6 | 10.2 | 0.9 | 6.7 | 0.8 |
| Liver | 19 | 3 | 10.5 | 0.5 | 10.5 | 0.4 | 9.5 | 0.4 | 9.1 | 0.3 | 7.5 | 0.4 |
| Muscle | 2.1 | 0.4 | 3.0 | 0.4 | 2.5 | 0.1 | 2.2 | 0.1 | 2.0 | 0.3 | 1.8 | 0.4 |
| Joint | 2.7 | 0.1 | 4.8 | 0.4 | 4.8 | 0.4 | 5.3 | 0.5 | 5.4 | 0.7 | 5.8 | 0.6 |

**Table S4-13.** ROI analysis of [^89^Zr]-DFO-RW03_scFv-Fc_ (1.40 ± 0.09 MBq, 16.0 ± 0.3 µg) co-injected with RW03_IgG_ (500 µg) (blocked). Uptake is expressed as mean percentage injected dose per unit volume (%ID/cc).

|  | 0.5 h | | 24 h | | 48 h | | 72 h | | 96 h | | 168 h | |
| --- | --- | --- | --- | --- | --- | --- | --- | --- | --- | --- | --- | --- |
|  | Mean | SEM | Mean | SEM | Mean | SEM | Mean | SEM | Mean | SEM | Mean | SEM |
| Tumor | 2.5 | 0.2 | 10 | 1 | 12.6 | 0.5 | 10.9 | 0.5 | 9.6 | 0.4 | 6.4 | 0.6 |
| Heart | 38 | 4 | 15.7 | 0.8 | 13.2 | 0.4 | 11.7 | 0.7 | 9.8 | 0.7 | 5.8 | 0.7 |
| Liver | 20 | 1 | 9.7 | 0.6 | 10.4 | 0.1 | 9.9 | 0.3 | 9.5 | 0.4 | 7.6 | 0.4 |
| Muscle | 2.0 | 0.2 | 3.0 | 0.2 | 2.4 | 0.1 | 2.4 | 0.3 | 2.1 | 0.2 | 1.5 | 0.2 |
| Joint | 2.4 | 0.2 | 4.5 | 0.3 | 4.3 | 0.2 | 4.3 | 0.1 | 4.5 | 0.5 | 4.5 | 0.2 |

**Table S4-14 Biodistribution table.** Ex vivo biodistribution (168 h p.i.) of [^89^Zr]-DFO-RW03_scFv-Fc_ (1.42 ± 0.07 MBq, 16.0 ± 0.2 µg) (Non-blocked), [^89^Zr]-DFO-RW03_scFv-Fc_ (1.40 ± 0.09 MBq, 16.0 ± 0.3 µg) co-injected with RW03_IgG_ (0.5 mg) (blocked). Uptake is expressed as mean percentage injected dose per gram tissue (%ID/g).

|  | Non-blocked | | | Blocked | | |
| --- | --- | --- | --- | --- | --- | --- |
|  | Mean | SEM | N | Mean | SEM | N |
| Blood | 6.3 | 0.9 | 5 | 5.1 | 0.6 | 5 |
| Adipose | 1.8 | 0.5 | 5 | 1.6 | 0.4 | 5 |
| Adrenals | 5 | 1 | 5 | 4.3 | 0.4 | 5 |
| Bone | 4.6 | 0.7 | 5 | 3.9 | 0.3 | 5 |
| Brain | 0.26 | 0.02 | 5 | 0.22 | 0.02 | 5 |
| Heart | 2.0 | 0.3 | 5 | 2.8 | 0.8 | 5 |
| Kidneys | 4.5 | 0.3 | 5 | 4.5 | 0.5 | 5 |
| L. Int + Caecum | 0.66 | 0.05 | 5 | 0.64 | 0.04 | 5 |
| Liver | 5.1 | 0.2 | 5 | 6.4 | 0.4 | 5 |
| Lungs | 5.0 | 0.4 | 5 | 3.4 | 0.7 | 5 |
| Pancreas | 1.1 | 0.1 | 5 | 3 | 2 | 5 |
| Skeletal Muscle | 0.7 | 0.1 | 5 | 1.6 | 0.8 | 5 |
| S. Int | 0.7 | 0.1 | 5 | 0.9 | 0.3 | 5 |
| Spleen | 4.9 | 0.9 | 5 | 7 | 3 | 5 |
| Stomach | 0.58 | 0.03 | 5 | 0.6 | 0.1 | 5 |
| Tumor | 18.2 | 0.7 | 5 | 5 | 1 | 5 |
| Urine + Bladder | 2.1 | 0.8 | 5 | 5 | 1 | 5 |

**Table S4-15. Biodistribution table.** Ex vivo biodistribution (168 h p.i.) of [^89^Zr]-DFO-RW03_scFv-Fc_ (1.42 ± 0.07 MBq, 16.0 ± 0.2 µg) (non-blocked), [^89^Zr]-DFO-RW03_scFv-Fc_ (1.40 ± 0.09 MBq, 16.0 ± 0.3 µg) co-injected with RW03_IgG_ (0.5 mg) (blocked). Uptake is expressed as mean percentage injected dose per gram tissue (%ID/O).

|  | Non-blocked | | | Blocked | | |
| --- | --- | --- | --- | --- | --- | --- |
|  | Mean | SEM | N | Mean | SEM | N |
| Blood | 5.6 | 0.7 | 5 | 4.4 | 0.6 | 5 |
| Adipose | 0.10 | 0.01 | 5 | 0.11 | 0.04 | 5 |
| Adrenals | 0.05 | 0.01 | 5 | 0.042 | 0.004 | 5 |
| Bone | 10 | 1 | 5 | 7.2 | 0.3 | 5 |
| Brain | 0.09 | 0.01 | 5 | 0.08 | 0.01 | 5 |
| Heart | 0.21 | 0.02 | 5 | 0.18 | 0.02 | 5 |
| Kidneys | 1.13 | 0.02 | 5 | 1.1 | 0.1 | 5 |
| L. Int + Caecum | 0.54 | 0.05 | 5 | 0.4 | 0.1 | 5 |
| Liver | 5.8 | 0.2 | 5 | 7.0 | 0.7 | 5 |
| Lungs | 0.64 | 0.05 | 5 | 0.39 | 0.03 | 5 |
| Pancreas | 0.15 | 0.04 | 5 | 0.3 | 0.2 | 5 |
| Skeletal Muscle | 5.2 | 0.5 | 5 | 6 | 1 | 5 |
| S. Int | 0.63 | 0.04 | 5 | 0.6 | 0.1 | 5 |
| Spleen | 0.44 | 0.03 | 5 | 0.7 | 0.3 | 5 |
| Stomach | 0.18 | 0.01 | 5 | 0.2 | 0.0 | 5 |
| Tumor | 4.1 | 0.7 | 5 | 2.2 | 0.5 | 5 |
| Urine + Bladder | 0.25 | 0.03 | 5 | 0.20 | 0.03 | 5 |
